# Immunotherapeutic potential of PD-1 blockade in chronic Leishmania mexicana infection through the enhancement of progenitor-like CXCR5^+^ and intermediate CXCR5^+^TIM-3^+^ exhausted T cells

**DOI:** 10.3389/fimmu.2026.1827753

**Published:** 2026-06-29

**Authors:** Mariana Diupotex, Julián A. Gajón, Laura C. Bonifaz, Jaime Zamora-Chimal, Ingeborg Becker

**Affiliations:** 1 Unidad de Investigación en Medicina Experimental, Facultad de Medicina, Universidad Nacional Autónoma de México, Ciudad de México, Mexico; 2 Instituto de Biotecnología, Universidad Nacional Autónoma de México, Campus Morelos, Cuernavaca, Morelos, Mexico; 3 Unidad de Investigación Médica en Inmunología, Hospital de Pediatría, Centro Médico Nacional Siglo XXI, Instituto Mexicano del Seguro Social, Ciudad de México, Mexico; 4 Unidad de Investigación Médica en Inmunoquímica, Hospital de Especialidades, Centro Médico Nacional Siglo XXI, Instituto Mexicano del Seguro Social, Ciudad de México, Mexico; 5 Coordinación de Investigación en Salud, Centro Médico Nacional Siglo XXI, Instituto Mexicano del Seguro Social, Ciudad de México, Mexico

**Keywords:** anti-PD-1, chronic infection, cutaneous leishmaniasis, immunotherapy, progenitor-like T cells, T-cell exhaustion, terminally-differentiated T cells

## Abstract

Diffuse cutaneous leishmaniasis (DCL), caused by *Leishmania mexicana*, is a chronic, treatment-refractory form of leishmaniasis characterized by multiple nodular lesions and high parasite load. During chronic infection, T cells acquire an exhausted immune state marked by elevated expression of PD-1 and comprise progenitor-like (CXCR5^+^TIM-3^-^), intermediate (CXCR5^+^TIM-3^+^), and terminally-differentiated (CXCR5^-^TIM-3^+^) exhausted T-cell (Tex) subsets. It remains unknown whether anti-PD-1 immune checkpoint therapy is effective during chronic *L. mexicana* infection and how it affects the Tex landscape. To address this, C57BL/6 mice chronically infected with *L. mexicana* were treated from day 45 post-infection with a variable-dose anti-PD-1 mAb regimen consisting of three 250 μg/dose induction doses followed by five 100 μg/dose maintenance doses. Anti-PD-1 immunotherapy limited lesion progression and reduced parasite burden in chronically infected mice. This effect was associated with enhanced antigen-specific Th1 immune response and functional reinvigoration of CD8^+^ and CD4^+^ T cells in draining lymph nodes, as evidenced by increased expression of CD69, Ki-67, IFN-γ, and GrzmB. Consistent with these findings, confocal microscopy analysis of footpad lesions revealed increased IFN-γ- and TNF-α-co-producing T cells. This study provides the first evidence that PD-1 blockade enhanced PD-1^+^CXCR5^+^ and PD-1^+^TIM-3^+^ Tex cells within lesion sites of *L. mexicana*-infected mice. In draining lymph nodes, anti-PD-1 treatment expanded progenitor-like CXCR5^+^TIM-3^-^ Tex cells in both CD4^+^ and CD8^+^ T-cell compartments, as well as intermediate CXCR5^+^TIM-3^+^ Tex cells within the CD8^+^ T-cell compartment. Overall, these findings demonstrate that anti-PD-1 immunotherapy improves clinical and parasitological outcomes during chronic *L. mexicana* infection in association with enhanced Th1-type immunity and remodeling of the exhausted T-cell compartment, supporting further investigation of PD-1/PD-L1 checkpoint blockade as a potential immunotherapeutic strategy for DCL.

## 1 Introduction

Immune checkpoint therapy (ICT) has emerged as a powerful strategy for cancer treatment, enhancing T cell-mediated immunity by targeting several inhibitory pathways. The programmed cell death protein (PD-1) receptor plays a pivotal role in immune regulation by binding to its ligands PD-L1 and PD-L2, thereby suppressing T-cell activity and evading immune surveillance (1, 2). Over the past decade, blockade of the PD-1/PD-L1 pathway has demonstrated remarkable clinical efficacy across multiple invasive malignancies, improving overall patient survival and achieving long-term disease control (3). PD-1/PD-L1-based monoclonal antibody (mAb) therapy has also been explored as a potential treatment for chronic infectious diseases, including leishmaniasis, as pathogens are known to exploit this inhibitory pathway to suppress host-protective antigen-specific immune responses (4).

Leishmaniasis refers to a group of neglected tropical vector-borne diseases caused by obligate protozoan parasites of the genus *Leishmania* (5). Clinical manifestations range from self-healing skin lesions to severe forms that metastasize to secondary sites via the lymphatic system (6). Anergic or diffuse cutaneous leishmaniasis (DCL) is a chronic, treatment-refractory form of the disease caused by *L. mexicana* in southeastern Mexico (7). It commonly manifests as multiple non-ulcerating papular to nodular lesions with high number of phagocytosed parasites, which coalesce into plaques resembling lepromatous leprosy (8). Unlike the benign localized cutaneous form, DCL follows a progressive course over months to years and, if left untreated, often results in significant scarring and disfigurement (9). Moreover, it has been associated with an increased risk of developing skin cancer, particularly cutaneous squamous cell carcinoma (cSCC) (10–12).

The current first-line treatment for DCL primarily relies on prolonged administration of pentavalent antimonials, either alone or in combination with other drugs such as sodium stibogluconate or miltefosine (13–15). However, this approach is not sustainable due to frequent relapses, clinical resistance, and potentially life-threatening side-effects (16). Moreover, pentavalent antimonials have a limited capacity to restore effective host immunity, which is particularly concerning given that patients with DCL exhibit a markedly impaired cellular immune response (7). Therefore, there is a critical need for therapies that not only target parasite clearance but also enhance or modulate protective immunity, enabling long-term infection control in patients with DCL (17).

Effective control of leishmaniasis requires a robust Th1/Tc1 response involving both CD4^+^ and CD8^+^ T cells. This response is characterized by the production of pro-inflammatory cytokines such as IFN-γ and TNF-α, which promote macrophage activation and intracellular parasite killing through inducible nitric oxide synthase (iNOS)-dependent mechanisms (18). However, persistent antigen exposure during chronic leishmaniasis progressively compromises T-cell-mediated immunity, ultimately leading to T-cell exhaustion, a distinct and epigenetically stable differentiation state characterized by sustained expression of inhibitory receptors, impaired proliferation, reduced cytokine production, and diminished cytolytic activity (19–23). Exhausted T (Tex) cells are now recognized as a dynamic, heterogeneous population composed of unique subsets exhibiting distinct therapeutic properties (24). In chronic viral infections and cancer, residual Tex cells with greater self-renewal and functional capacity have been associated with responsiveness to PD-1 blockade, whereas more terminally exhausted populations exhibit limited responsiveness to ICT (25–27).

Recently, we demonstrated that in experimental chronic leishmaniasis caused by *L. mexicana*, PD-1^+^ Tex cells comprise three operationally defined subsets based on the expression of CXCR5 and TIM-3: progenitor-like (CXCR5^+^TIM-3^-^), intermediate (CXCR5^+^TIM-3^+^), and terminally-differentiated (CXCR5^-^TIM-3^+^) cells. Progenitor-like Tex cells exhibited enhanced proliferative capacity and IFN-γ production together with lower expression of inhibitory receptors, whereas intermediate Tex cells retained proliferative, cytotoxic, and IFN-γ-producing capabilities. In contrast, terminally-differentiated Tex cells displayed elevated expression of inhibitory receptors associated with reduced proliferative capacity and IFN-γ production (28). Previous studies have shown that blockade of the PD-1/PD-L1 axis can partially restore T-cell effector functions and reduce parasite burden in experimental leishmaniasis caused by *L. donovani* or *L. amazonensis* (29, 30). However, the cellular mechanisms underlying the response to PD-1-targeted therapy remain poorly understood, and the therapeutic efficacy of PD-1 blockade during chronic *L. mexicana* infection has not yet been established. Identifying the Tex cell populations associated with responsiveness to ICT may improve our understanding of immune control during chronic leishmaniasis. Therefore, the aim of this study was to evaluate the effect of anti-PD-1 mAb therapy in experimental chronic leishmaniasis caused by *L. mexicana* and to identify the exhausted Tex subset responsive to anti-PD-1 therapy.

## 2 Results

### 2.1 Anti-PD-1 immunotherapy limits clinical and parasitological disease progression

To explore the therapeutic potential of PD-1 blockade in experimental chronic leishmaniasis, anti-PD-1 immunotherapy was evaluated in *L. mexicana*-infected mice. In an initial approach, mice with established infection received ten intraperitoneal administrations of anti-PD-1 mAb at 100 μg/dose (4.5 mg/kg) every 3 days, following a regimen previously shown to reduce parasite burden in experimental *L. amazonensis* infection (30). In the chronic *L. mexicana* infection model, this fixed-dose regimen showed limited therapeutic efficacy, resulting in a modest reduction in lesion size without significantly affecting parasite burden (**Supplementary Figure 1**).

**Figure 1:**
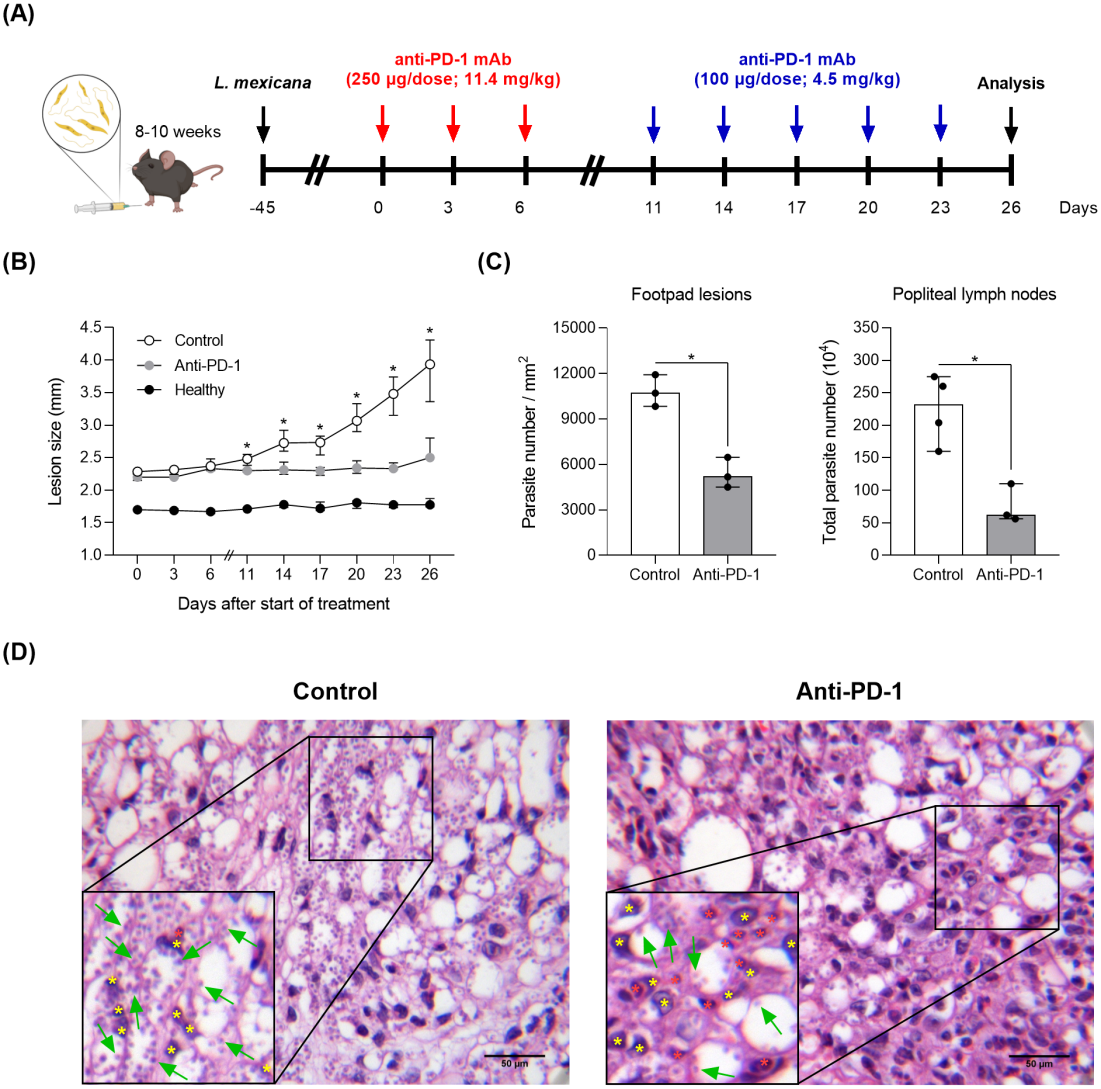
Anti-PD-1 immunotherapy reduces lesion development and parasite load in chronic *L. mexicana* infection. **(A)** C57BL/6 mice were subcutaneously infected with 1x10^5^ stationary-phase promastigotes and treated intraperitoneally starting at day 45 post-infection with anti-PD-1 mAb, administered at 250 μg per dose on days 0, 3, and 6, followed by a 5-day rest period and five additional doses of 100 μg per dose on days 11, 14, 17, 20, and 23. **(B)** Lesion progression was monitored by measuring footpad thickness with digital vernier caliper. Each point represents the median value obtained from 3-4 independently analyzed mice, with error bars indicating the full range (min -max). Asterisks indicate significant differences between untreated and anti-PD-1-treated mice. **(C)** Parasite load in footpad lesions and popliteal draining lymph nodes (dLNs) was quantified at the end of treatment. Each dot represents one independently analyzed mouse (n=3-4), and bars indicate the median and full range (min-max). **(D)** Representative H&E-stained sections of footpad tissue from untreated and anti-PD-1 -treated mice. Green arrowheads indicate intracellular amastigotes, whereas yellow and red asterisks show infected macrophages and inflammatory cell infiltration, respectively. All statistical analyses were performed using Mann-Whitney U-test. *P ≤ 0.05. Created in https://BioRender.com.

To improve the therapeutic efficacy observed with the fixed-dose protocol, an empirically optimized variable-dosing anti-PD-1 regimen consisting of 3 administrations of anti-PD-1 mAb at 250 μg/dose (11.4 mg/kg) every 3 days followed by 5 administrations at 100 μg/dose (4.5 mg/kg) every 3 days was subsequently evaluated (**Figure 1A**). The dosing scheme employed in this regimen was designed based on two lines of evidence. First, approved clinical anti-PD-1 therapies (nivolumab, pembrolizumab) consistently use an induction phase with higher or more frequent doses followed by a lower-dose maintenance phase, a strategy supported by pharmacokinetic modeling demonstrating the importance of achieving and sustaining PD-1 receptor saturation early in treatment (31, 32). Second, previous murine studies of anti-PD-1 in *Leishmania* infection demonstrated that a single weekly dose of 100 µg was insufficient to reduce parasite burden, highlighting the need for higher induction doses to achieve effective PD-1 blockade in this chronic infection context (30). Accordingly, we administered three induction doses of 250 µg to ensure rapid and complete PD-1 saturation, followed by maintenance doses of 100 µg to sustain blockade while minimizing potential toxicity.

Under variable-dose regimen, anti-PD-1–treated mice developed significantly smaller lesions than control animals starting on day 11 of treatment. Interestingly, lesion size in treated mice remained stable throughout the follow-up, whereas lesions in control mice increased progressively over time. Compared to controls, treated mice showed lower lesion thickness, with differences of -0.18 mm on day 11 (CI90%: -0.27 to -0.06), -0.40 mm on day 14 (CI90%: -0.68 to -0.17), -0.44 mm on day 17 (CI90%: -0.60 to -0.19), -0.71 mm on day 20 (CI90%: -1.07 to -0.45), -1.11 mm on day 23 (CI90%: -1.42 to -0.73), and -0.86 mm on day 26 (CI90%: -1.81 to -0.48), highlighting the sustained therapeutic effect of anti-PD-1 treatment (**Figure 1B**). In addition, no reduction in body weight was observed during mAb administration (data not shown). Parasite burden in lesion sites and popliteal lymph nodes was significantly reduced, by at least 2-fold, in anti-PD-1–treated mice compared to control animals. The median difference between treated and control mice was -5,451 parasites (CI90%: -7,410 to -3,364) in the footpad and -149 parasites (1x10^4^) (CI90%: -219 to -50) in the lymph nodes (**Figure 1C**). Histopathological analysis of footpad tissue revealed a marked reduction in the number of intracellular parasites, along with an increased inflammatory infiltrate in anti-PD-1 treated mice (**Figure 1D**). Overall, these results indicate that anti-PD-1 immunotherapy, administered under a variable-dose regimen, limits lesion progression and parasite load in chronic *L. mexicana* infection.

### 2.2 PD-1 blockade enhances Th1-type immune responses

**Figure 2:**
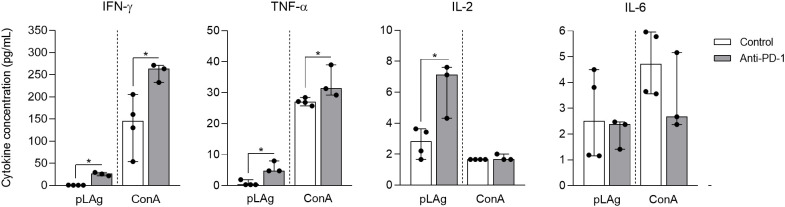
PD-1 checkpoint blockade enhances pro-inflammatory cytokine production in response to leishmanial antigens. Cells from popliteal dLNs of untreated or anti-PD-1–treated mice were stimulated with promastigote antigen (pLAg) or Concanavalin A (ConA), and cytokine production was evaluated in culture supernatants by flow cytometry. Each dot represents one independently analyzed mouse. Data are presented as median with range (min–max) from 3–4 mice per experimental group. All statistical analyses were performed using Mann-Whitney U-test. *P ≤ 0.05.

We next asked whether the improved clinical and parasitological outcome was associated with enhanced antigen-specific Th1 responses in draining lymph nodes (dLNs). To address this, Th1/Th2 cytokine production by popliteal dLNs cells was assessed following stimulation with promastigote antigen (pLAg) to evaluate antigen-specific cytokine responses, or with the mitogen concanavalin A (ConA) to assess overall T cell functionality. Anti-PD-1 treatment significantly enhanced the production of proinflammatory cytokines under both stimulation conditions. Following pLAg stimulation, IFN-γ, TNF-α, and IL-2 concentrations were markedly higher in anti-PD-1–treated mice compared to control groups, with median differences of 25.39 pg/mL (CI94.29%: 20.85 to 28.79), 4.46 pg/mL (CI94.29%: 2.84 to 7.67), and 3.83 pg/mL (CI94.29%: 0.68 to 5.95), respectively. In response to ConA stimulation, a similar proinflammatory shift was observed, with a difference of 107.2 pg/mL (CI94.29%: 27.09 to 217.60) in IFN-γ levels and 4.32 pg/mL (CI94.29%: 0.78 to 13.23) in TNF-α production. No differences in IL-2 production were detected between treated and control mice following ConA stimulation. IL-6 production also showed no differences under any analyzed conditions (**Figure 2**). In contrast, the production of anti-inflammatory Th2-associated cytokines (IL-4, IL-5, IL-10, and IL-13) remained comparable between treated and untreated mice under both stimulation conditions (data not shown). These findings indicate that anti-PD-1 immunotherapy promotes a proinflammatory Th1-associated immune response during chronic *L. mexicana* infection, characterized by enhanced production of IFN-γ, TNF-α, and IL-2.

### 2.3 Anti-PD-1 therapy reinvigorates T-cell responses at the lesion sites and lymph nodes

**Figure 3:**
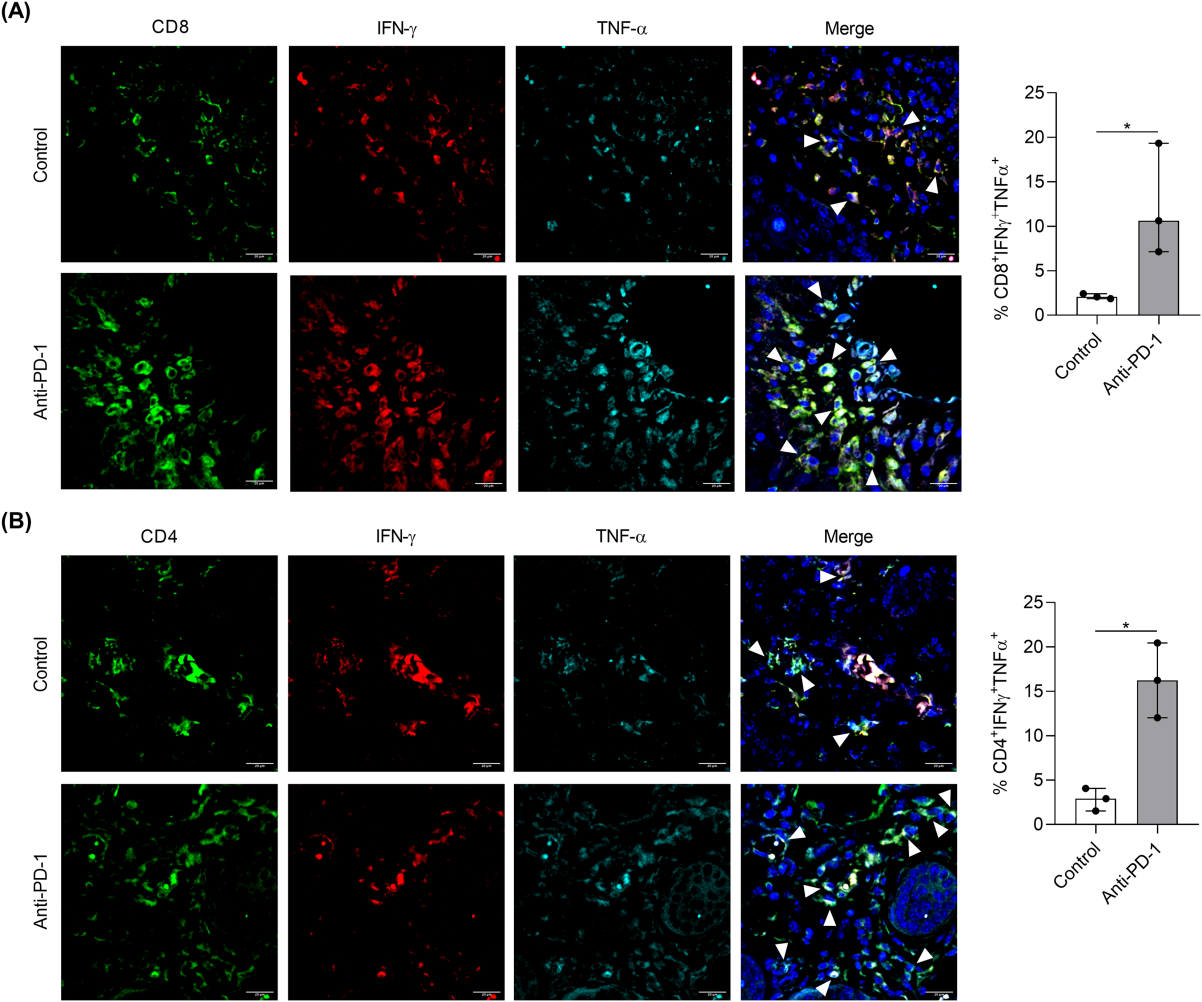
PD-1/PD-L1 blockade enhances IFN-γ and TNF-α production by T lymphocytes in lesions. Footpad sections from untreated or anti-PD-1 treated mice were immunostained with anti-CD4, anti-CD8, anti-IFN-γ, and anti-TNF-α antibodies. Nuclei were counterstained with Hoechst 33258. Confocal microscopy images show cytokine expression within CD8^+^ **(A)** or CD4^+^ **(B)** T cells. White arrowheads indicate cells co-expressing IFN-γ and TNF-α. Each dot represents one independently analyzed mouse. Data are presented as median with range (min–max) from 3–4 mice per experimental group. All statistical analyses were performed using Mann-Whitney U-test. *P ≤ 0.05.

To determine whether anti-PD-1 therapy restored T-cell effector responses during chronic infection, T-cell activation, proliferation, and effector functions were evaluated at the primary (footpad lesions) and secondary (dLNs) sites of infection. A significantly higher frequency of CD8^+^IFN-γ^+^TNF-α^+^ (**Figure 3A**) and CD4^+^IFN-γ^+^TNF-α^+^ cells (**Figure 3B**) was detected in the lesions of anti-PD-1–treated mice compared to untreated animals, with median differences of 8.55% (CI90%: 4.72 to 17.47) and 13.33% (CI90%: 7.97 to 18.93), respectively. These findings indicate that anti-PD-1 immunotherapy increases local CD8^+^ and CD4^+^ T cells coexpressing IFN-γ and TNF-α.

**Figure 4:**
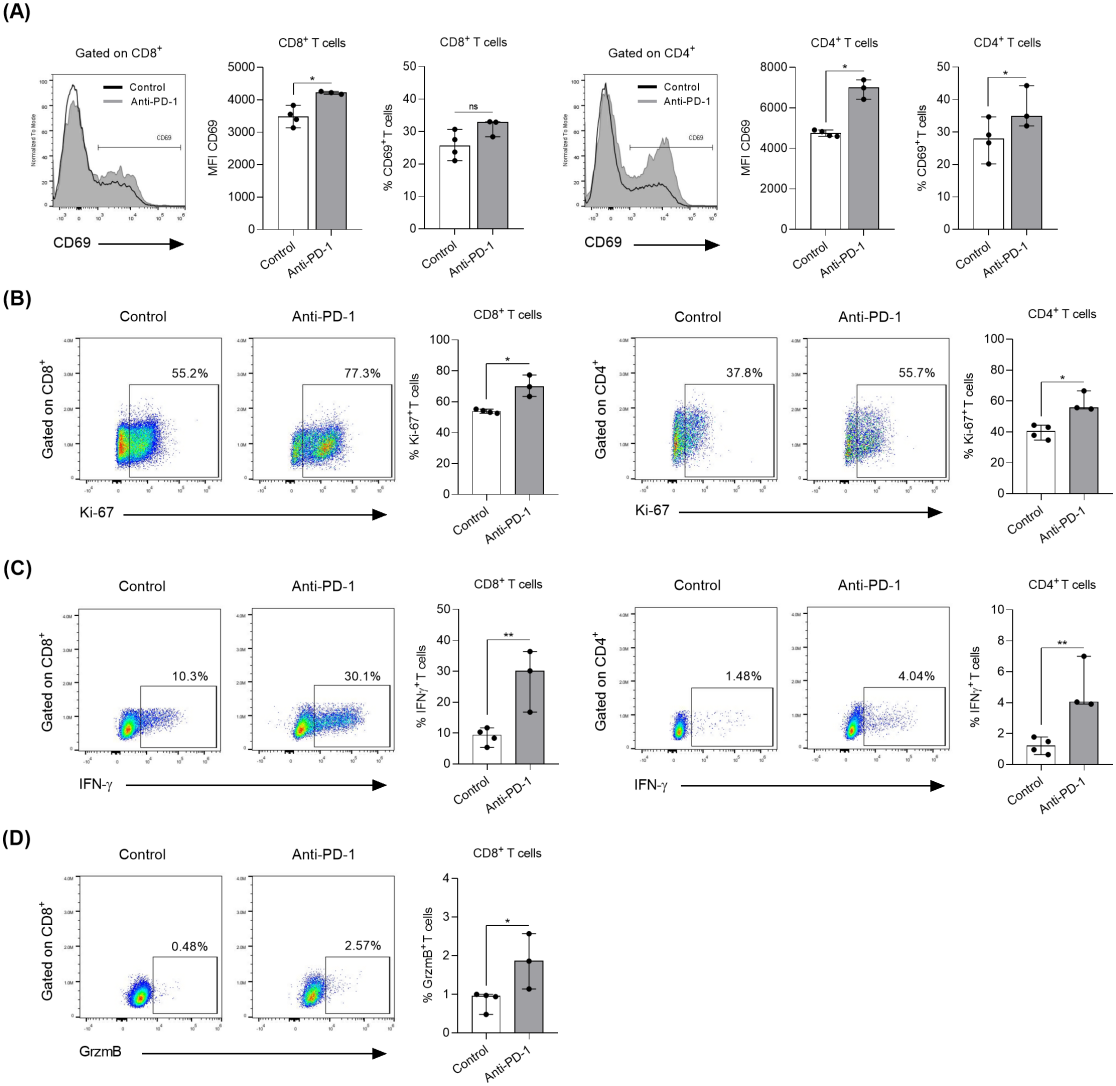
PD-1 blockade enhances T-cell activation and effector responses in secondary lymphoid tissues. Popliteal lymph node cells of untreated or anti-PD-1–treated mice, either freshly isolated or polyclonally stimulated with ConA or PMA/Ionomycin, were analyzed by flow cytometry. Representative histograms or dot plots show the expression of CD69 **(A)**, Ki-67 **(B)**, IFN-γ **(C)**, and GrzmB **(D)** in CD8^+^ and CD4^+^ T cells. Each dot represents one independently analyzed mouse. Data are presented as median with range (min–max) from 3–4 mice per experimental group. All statistical analyses were performed using Mann-Whitney U-test. *P ≤ 0.05, **P ≤ 0.01.

Freshly isolated CD8^+^ and CD4^+^ T cells from dLNs of anti-PD-1–treated mice exhibited higher expression of the activation marker CD69 compared to untreated controls, with MFI differences of 2,253 (CI94.29%: 1514 to 2784) and 739 (CI94.29%: 343 to 1122) for CD4^+^ and CD8^+^ T cells, respectively. The frequency of CD69^+^ cells within the CD8^+^ T-cell compartment did not differ significantly between groups. In contrast, a significantly higher proportion of CD69^+^ cells was observed among CD4^+^ T cells in treated mice, showing a difference of 8.90% (CI94.29%: -2.80 to 24.10) (**Figure 4A**). Furthermore, the proportion of actively proliferating Ki-67^+^ cells within the CD8^+^ T-cell compartment was significantly higher in anti-PD-1–treated mice, with a median difference of 16% (CI94.29%: 8.20 to 24.80). Similarly, the CD4^+^ T-cell compartment from treated mice also exhibited a higher proportion of Ki-67^+^ proliferating cells, with a median difference of 19.05% (CI94.29%: 10.60 to 31.80) compared to controls (**Figure 4B**). Consistent with these results, a higher frequency of IFN-γ^+^ cells was detected in both CD8^+^ and CD4^+^ T-cell compartments from anti-PD-1–treated mice, with median differences of 20.77% for CD8^+^ T cells (CI94.29%: 5.10 to 31.04) and 3.17% for CD4^+^ T cells (CI94.29%: 2.13 to 6.36) (**Figure 4C**). No significant differences were observed in the proportion of TNF-α-producing CD8^+^ or CD4^+^ T cells between groups (data not shown). Finally, anti-PD-1 therapy induced a modest but significant increase in the frequency of CD8^+^GrzmB^+^ T cells, with a median difference of 0.90% (CI94.29%: 0.14 to 2.09) compared to controls (**Figure 4D**), whereas no significant differences were detected in the frequency of CD8^+^CD107a^+^ cells between treated and untreated animals (data not shown). Collectively, these data indicate that anti-PD-1 immunotherapy reinvigorates T-cell responses at both lesion sites and lymph nodes during chronic *L. mexicana* infection, promoting enhanced activation, proliferation, and effector functions.

### 2.4 Progenitor-like CXCR5^+^TIM-3^-^ and intermediate CXCR5^+^TIM-3^+^ Tex subsets selectively expand after PD-1 checkpoint blockade

**Figure 5:**
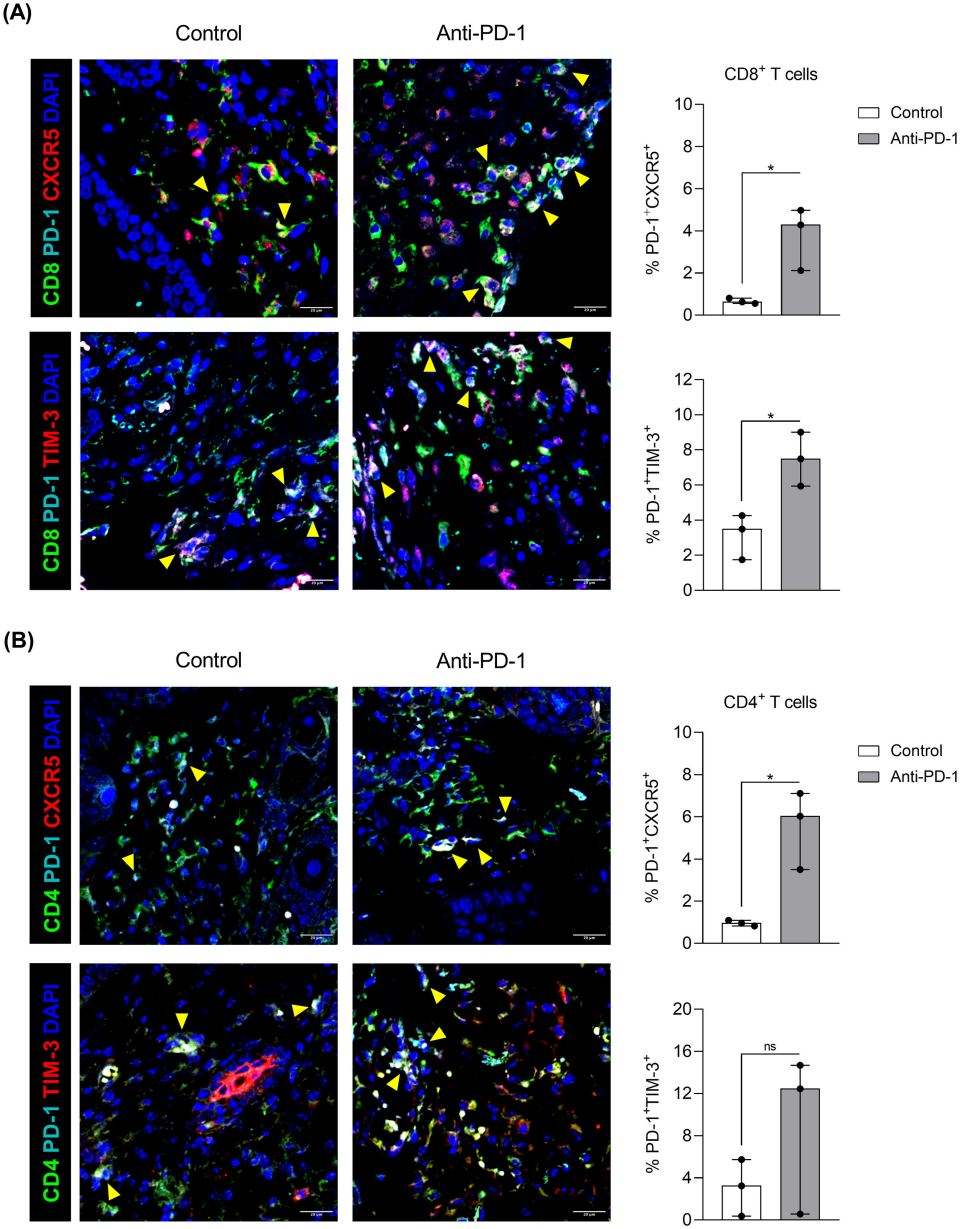
PD-1^+^CXCR5^+^ or PD-1^+^TIM-3^+^ T cells increase in lesions following PD-1 checkpoint blockade. Footpad sections from untreated or anti-PD-1–treated mice were immunostained with anti-CD4, anti-CD8, anti-PD-1, anti-CXCR5 or anti-TIM-3 antibodies. Nuclei were counterstained with Hoechst 33258. Yellow arrowheads in representative confocal microscopy images indicate co-expression of PD-1 and CXCR5 or PD-1 and TIM-3 within CD8^+^ **(A)** and CD4^+^ **(B)** T cells. Each dot represents one independently analyzed mouse. Data are presented as median with range (min–max) from 3–4 mice per experimental group. All statistical analyses were performed using Mann-Whitney U-test. *P ≤ 0.05. ns indicates not significant.

Since anti-PD-1 therapy reinvigorated CD8^+^ and CD4^+^ T-cell responses, we next investigated which of the previously described Tex subsets (28) were associated with responsiveness to PD-1 blockade in chronic *L. mexicana* infection. To identify progenitor-like (CXCR5^+^TIM-3^−^) and terminally-differentiated (CXCR5^−^TIM-3^+^) Tex cells within lesion sites, exhausted PD-1^+^ cells were evaluated for the expression of lymphoid tissue-homing receptor CXCR5 and exhaustion-associated receptor TIM-3. Anti-PD-1 treatment led to higher frequencies of progenitor-like PD-1^+^CXCR5^+^ and terminally-differentiated PD-1^+^TIM-3^+^ Tex cells within the CD8^+^ T-cell compartment compared with untreated controls, exceeding them by 3.60% (CI90%: 1.31 to 4.42) and 4.18% (CI90%: 1.67 to 7.25), respectively (**Figure 5A**). In the CD4^+^ T-cell compartment, anti-PD-1–treated mice showed an increase in progenitor-like PD-1^+^CXCR5^+^ Tex cells, with a median difference of 5.07% (CI90%: 2.40 to 6.28) compared to untreated control, whereas no significant differences were observed in the frequency of terminally-differentiated PD-1^+^TIM-3^+^ Tex cells between groups (**Figure 5B**). These findings indicate that anti-PD-1 immunotherapy is preferentially associated with an increase in progenitor-like Tex cells within lesion sites during chronic *L. mexicana* infection.

**Figure 6:**
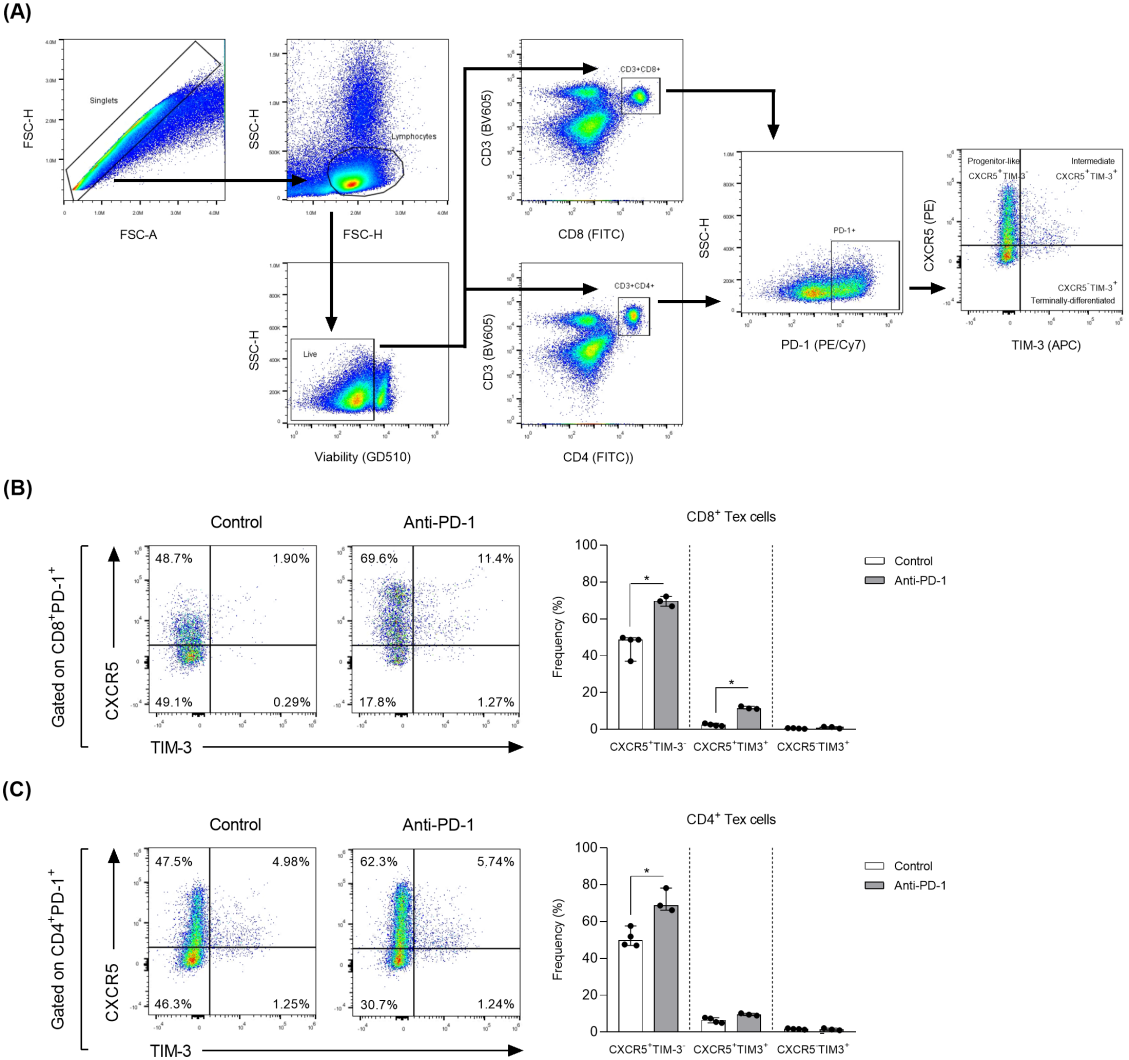
Progenitor-like CXCR5^+^ and intermediate CXCR5^+^TIM-3^+^ Tex cells expand in secondary lymphoid tissues following anti-PD-1 treatment. Freshly isolated popliteal lymph node cells from untreated or anti-PD-1–treated mice were immunostained for flow cytometry analysis. **(A)** Single events were gated based on forward scatter (FSC-H vs FSC-A), and lymphocytes were identified according to size and granularity (SSC-H vs. FSC-H). Dead cells were excluded, and viable cells were gated as CD3^+^CD4^+^ or CD3^+^CD8^+^ T cells. Tex subsets were defined as PD-1^+^ T cells and further categorized according to CXCR5 and TIM-3 expression. The frequency of each Tex subset within CD8^+^ **(B)** and CD4^+^ **(C)** T cells was determined. Each dot represents one independently analyzed mouse. Data are presented as median with range (min–max) from 3–4 mice per experimental group. All statistical analyses were performed using Mann-Whitney U-test. *P ≤ 0.05.

To achieve a more detailed resolution of Tex subsets, including the identification of the intermediate (CXCR5^+^TIM-3^+^) Tex cells, flow cytometry analysis was performed on freshly isolated popliteal dLNs cells (**Figure 6A**). Anti-PD-1 treatment significantly increased the frequency of CD8^+^ progenitor-like CXCR5^+^TIM-3^-^ and intermediate CXCR5^+^TIM-3^+^ Tex cells compared to the control group, with estimated differences of 21.65% (CI94.29%: 17.10 to 35.20) and 9.21% (CI94.29%: 7.82 to 10.60), respectively. No significant differences were observed in the proportion of CD8^+^ terminally-differentiated CXCR5^-^TIM-3^+^ Tex cells between groups (**Figure 6B**). In the CD4^+^ T-cell compartment, progenitor-like CXCR5^+^TIM-3^-^ Tex cells were markedly more abundant in anti-PD-1–treated mice, exceeding control values by 19.90% (CI94.29%: 8.60 to 21.20), whereas intermediate CXCR5^+^TIM-3^+^ and terminally-differentiated CXCR5^-^TIM-3^+^ Tex subsets remained comparable between groups (**Figure 6C**). Overall, these results show that anti-PD-1 treatment promotes the increase of progenitor-like and intermediate CD8^+^ Tex subsets in the dLNs, whereas in the CD4^+^ T-cell compartment, the increase was restricted to the progenitor-like Tex population, mirroring the pattern observed in the lesion sites.

## 3 Discussion

Immune checkpoint inhibitors (ICIs) have transformed the therapeutic landscape of cancer and are now a standard treatment option alongside surgery, radiotherapy, and chemotherapy. Blockade of the PD-1/PD-L1 pathway has shown remarkable clinical benefits, extending the overall survival of patients with advanced melanoma to approximately 5 years as monotherapy and up to 6.5 years when combined with other ICIs (33, 34). The benefits of this approach are not limited to oncology, as several studies have demonstrated the efficacy of PD-1/PD-L1 blockade in chronic infectious diseases (35). In this study, we evaluated the immunotherapeutic potential of a PD-1-blocking mAb in an experimental chronic infection caused by *L. mexicana*, the etiological agent of DCL in Mexico.

To assess the therapeutic response to PD-1 blockade, the mAb was administered to chronically infected C57BL/6 mice. This model was selected due to its inability to resolve *L. mexicana* infection despite the mice’s genetic predisposition to establish a Th1-type immune response (36, 37). Importantly, this chronic infection model reproduces several immunopathological features observed in patients with DCL, including persistent lesions, high parasite burden, impaired Th1-type cytokine production, and the presence of functionally exhausted T-cell responses (28). We first tested a treatment regimen based on that reported by da Fonseca-Martins et al. in early infection caused by *L. amazonensis* (30). In our protocol, anti-PD-1 mAb was administered intraperitoneally starting 52 days post-infection, at ten doses of 100 μg every three days. Under this fixed-dose regimen, lesions were slightly smaller, while no significant differences in parasite load were observed. Although this protocol provided some evidence of clinical effects, no meaningful therapeutic impact was achieved. Our findings differ from those of the original study, which reported a marked reduction in parasite load when treatment was initiated at day 7 post-infection, although lesion size remained largely unaffected (30). These findings underscore the need for more tailored therapeutic strategies, since the effectiveness of ICIs may be strongly influenced by both the stage of disease and the infecting species.

PD-1/PD-L1 blockade in clinical oncology is commonly administered at fixed, regular intervals, either as monotherapy or in combination with other ICIs (38). However, alternative two-phase treatment strategies have been explored to rapidly prime the immune system during an induction phase aimed at achieving complete or partial remission, followed by a lower-intensity maintenance phase designed to sustain immune responses, minimize toxicity, and prolong progression-free survival (39). These induction/maintenance approaches have shown improved therapeutic efficacy in different cancer settings, including melanoma and non-small cell lung cancer (34, 40, 41). Based on this rationale, an empirically optimized variable-dose regimen incorporating both high and lower anti-PD-1 doses was evaluated in our chronic infection model to improve the therapeutic efficacy of anti-PD-1 blockade.

This second regimen was initiated 45 days post-infection, when lesion size was comparable to that of mice at the onset of the first treatment protocol. In this regimen, anti-PD-1 mAb was administered intraperitoneally as three induction doses of 250 μg every three days, followed by five maintenance doses of 100 μg at the same interval. This variable-dosing regimen resulted in a more favorable therapeutic response compared to the fixed-dose protocol. Notably, lesion size did not increase throughout treatment, and parasite burden was markedly reduced in both lesion sites and lymph nodes, underscoring the therapeutic potential of anti-PD-1 immunotherapy for chronic *L. mexicana* infection.

Our findings are consistent with previous studies demonstrating that PD-1/PD-L1 blockade reduces parasite load in experimental infections with *L. donovani* and *L. amazonensis* (29, 30). In those studies, however, treatment was initiated very early, before the infection became established, and thus did not evaluate therapy in the chronic phase of the disease. To the best of our knowledge, this is the first study to demonstrate the efficacy of anti-PD-1 mAb therapy in a chronic *L. mexicana* infection. Moreover, it is the first to implement an alternative immunotherapeutic regimen with high induction doses of anti-PD-1 mAb therapy followed by lower maintenance doses, demonstrating efficacy at both clinical and parasitological levels. Although this variable-dose strategy was empirically optimized within the chronic *L. mexicana* infection model, the therapeutic efficacy observed highlights the importance of dosing strategy in optimizing checkpoint blockade therapies, particularly in chronic parasitic diseases where the immunological context differs from that of cancer. Therefore, the optimal dosing strategy, duration of PD-1 blockade, and minimum effective dose required to maximize therapeutic efficacy while limiting potential toxicity, remain to be determined. Future studies incorporating pharmacokinetic/pharmacodynamic (PK/PD)-guided optimization will be important to refine ICT strategies for chronic parasitic infections.

Since the variable-dosing regimen limited lesion progression and reduced parasite load, we next assessed its effect on Th1- and Th2-type cytokine production in supernatants from lymph node cells. Our results showed that anti-PD-1 immunotherapy enhances both antigen-specific and non-specific production of IFN-γ, TNF-α, and IL-2, indicating that PD-1/PD-L1 blockade promotes proinflammatory Th1-associated immune responses during chronic infection. This cytokine profile is particularly relevant in leishmaniasis, since IFN-γ drives CD4^+^ Th1 differentiation and activates phagocytes to eliminate intracellular parasites, while TNF-α further reinforces classical macrophage activation, leukocyte recruitment, and granuloma formation (42–44). In parallel, IL-2 supports the proliferation and survival of antigen-specific T cells and further amplifies IFN-γ production (45). Its upregulation in our infection model also supports effective engagement of the PD-1 receptor by the anti-PD-1 antibody, consistent with reports that IL-2 production reflects successful blockade (39).

ICIs differ from conventional anticancer drugs by acting through indirect mechanisms, as blockade of the PD-1/PD-L1 axis promotes tumor rejection primarily through reinvigoration of T-cell mediated immunity (46). In line with this, our results show that anti-PD-1 mAb therapy increases the frequency of IFN-γ and TNF-α coexpressing T cells at lesion sites in both CD8^+^ and CD4^+^ T-cell compartments. The increase in these polyfunctional T cells indicates improved local effector responses, which may contribute to the reduced parasite burden and limited lesion progression observed following treatment. Indeed, IFN-γ and TNF-α are widely recognized to act synergistically to enhance nitric oxide production and promote macrophage leishmanicidal activity, underscoring the capacity of PD-1 blockade to reinvigorate local T-cell responses in chronic infection by *L. mexicana* (47).

Immunotherapy also promoted T-cell activation in draining lymph nodes, as evidenced by increased CD69 expression in both CD8^+^ and CD4^+^ T-cell compartments. Furthermore, elevated frequencies of actively dividing Ki-67^+^ cells and IFN-γ-producing cells were detected within both T-cell compartments following therapy. Interestingly, a slight increase in GrzmB-expressing cells was also observed following anti-PD-1 therapy; however, no differences were detected in CD107a expression, indicating that PD-1 blockade during chronic *L. mexicana* infection did not induce a marked restoration of cytotoxic CD8^+^ T-cell responses. Instead, the most prominent immunological changes associated with immunotherapy were the increased of IFN-γ-producing CD8^+^ and CD4^+^ T cells. These findings are consistent with previous studies showing that blockade of PD-1/PD-L1 signaling enhances proinflammatory cytokine production by both CD4^+^ and CD8^+^ T cells during experimental *L. donovani* and *L. amazonensis* infections (29, 30). Collectively, these data indicate that the therapeutic efficacy of PD-1 blockade during leishmaniasis is associated predominantly with reinvigoration of Th1-type immune responses.

The clinical efficacy of PD-1/PD-L1 immunotherapy was initially attributed to the reinvigoration of all Tex cells (46). However, it is now recognized that therapeutic success in chronic viral infections and cancer does not rely on the entire exhausted pool, but rather on precursor-like (TCF1^+^) and transitory-effector CD8^+^ Tex cells (48). Recently, we demonstrated that during chronic *L. mexicana* infection, CD4^+^ and CD8^+^ PD-1^+^ Tex cells comprise three distinct subsets (1): progenitor-like CXCR5^+^TIM-3^-^ cells, displaying higher proliferative capacity and IFN-γ production, associated with the lowest expression of inhibitory receptors; (2) intermediate CXCR5^+^TIM-3^+^ cells, which retain proliferative, cytotoxic and cytokine-mediated effector functions; and (3) terminally-differentiated CXCR5^-^TIM-3^+^ cells, characterized by the highest expression of inhibitory receptors and reduced proliferation and IFN-γ production (28). As we observed that anti-PD-1 mAb therapy reinvigorates effector functions in both CD8^+^ and CD4^+^ T cells, we sought to identify the specific Tex subset targeted by anti-PD-1 mAb therapy during chronic *L. mexicana* infection.

Our results demonstrated that progenitor-like CXCR5^+^TIM-3^-^ Tex cells within both CD8^+^ and CD4^+^ T-cell compartments, as well as intermediate CXCR5^+^TIM-3^+^ Tex cells within the CD8^+^ T-cell compartment, were increased in lymph nodes after anti-PD-1 mAb therapy, suggesting that these subsets could be related to the beneficial outcomes of the immunotherapy. These results are consistent with previous findings in chronic lymphocytic choriomeningitis virus (LCMV) infection showing that PD-1 blockade expands precursor-like TCF1^+^CD8^+^ Tex cells, a subset phenotypically and functionally similar to the progenitor-like Tex cells identified in our chronic infection model (28, 49, 50). Importantly, our findings further demonstrated that anti-PD-1 immunotherapy increases both CD8^+^ and CD4^+^ progenitor-like CXCR5^+^TIM-3^-^ Tex cells, indicating that responsiveness to PD-1 blockade during chronic *L. mexicana* infection is not restricted to CD8^+^ Tex cells. Despite the predominant focus on CD8^+^ Tex responses in the immunotherapy field, previous studies have shown that anti-PD-L1 treatment increases TCF1 expression while reducing TIM-3 and LAG-3 expression in CD4^+^ T cells from B16.F10-tumor bearing mice, supporting the maintenance of precursor-like TCF1^+^CD4^+^ T cells and contributing to improved tumor growth control (51). Accordingly, we hypothesize that progenitor-like CXCR5^+^TIM-3^-^ CD4^+^ Tex cells expanded in our infection model contribute, together with progenitor-like CXCR5^+^TIM-3^-^ CD8^+^ Tex cells, to infection control following immunotherapy.

The differential expansion pattern observed between CD8^+^ and CD4^+^ Tex cells following PD-1 blockade may reflect intrinsic differences in their differentiation programs. Recent studies using longitudinal single-cell transcriptomic and TCR sequencing analyses have shown that ICT induces distinct waves of clonal Tex responses with different temporal kinetics and differentiation trajectories (52). In line with this, the more restricted expansion observed in CD4^+^ Tex cells following PD-1 blockade, limited to progenitor-like CXCR5^+^TIM-3^-^ Tex subset, is consistent with the current understanding that CD4^+^ and CD8^+^ T-cell exhaustion represent fundamentally divergent processes. Indeed, previous studies demonstrated that terminal exhaustion in CD4^+^ tumor-infiltrating lymphocytes is established independently of TIM-3 expression, in contrast to the hierarchical CD8^+^ Tex differentiation program characterized by the progressive acquisition of TIM-3 expression (53). Additional studies will be required to determine whether intermediate CXCR5^+^TIM-3^+^ CD8^+^ Tex cells exhibit greater responsiveness to immunotherapy than their CD4^+^ counterparts, or whether differentiation trajectories following PD-1 blockade fundamentally differ according to T-cell lineage.

Previous studies have shown that PD-1 blockade reinvigorates and expands precursor-like TCF1^+^ Tex cells localized within lymphoid tissues or the tumor microenvironment. These Tex subsets sustain long-term T-cell responses through asymmetric division, thereby maintaining the precursor-like pool while simultaneously generating transitory-effector progeny capable of migrating to peripheral tissues. In contrast, terminally-differentiated TCF1^-^ Tex cells exhibit reduced responsiveness to checkpoint blockade, as they display poor self-renewal and diminished effector functions (49, 50, 54). In line with these observations, we hypothesize that preexisting progenitor-like CXCR5^+^TIM-3^-^ Tex cells of our chronic infection model represent a functionally responsive compartment that is reinvigorated upon anti-PD-1 mAb therapy. In this context, PD-1 blockade may promote the expansion of progenitor-like CXCR5^+^TIM-3^-^ Tex cells within lymphoid tissues, followed by their differentiation into intermediate effector subsets capable of migrating to infected lesions through the CX3CR1-CX3CL1 axis. This hypothesis is consistent with our previous findings showing that progenitor-like CXCR5^+^TIM-3^-^ Tex cells exhibit high proliferative capacity and elevated CCR7 expression, whereas intermediate CXCR5^+^TIM-3^+^ Tex cells display the highest expression of CX3CR1 (28).

The beneficial clinical and parasitological effects observed following immunotherapy may be linked to the preserved effector functions of progenitor-like and intermediate Tex subsets despite maintaining exhaustion-associated features. Indeed, our previous study demonstrated that progenitor-like CXCR5^+^TIM-3^-^ and intermediate CXCR5^+^TIM-3^+^ Tex subsets retain greater proliferative capacity and IFN-γ production compared with terminally-differentiated CXCR5^-^TIM-3^+^ Tex cells, which display a more dysfunctional phenotype (28). These findings are consistent with our current observations showing increased frequencies of IFN-γ-producing cells following PD-1 blockade. However, it remains to be determined whether progenitor-like and intermediate Tex subsets directly or indirectly mediate parasite control. Addressing this question will require further functional approaches such as subset-specific depletion or adoptive transfer experiments, to establish the contribution of these populations during anti-PD-1 therapy.

Although the heterogeneity of Tex cells in human CL remains incompletely defined, our previous work demonstrated that CD8^+^ T cells from patients with DCL retain residual effector activity and exhibit partial functional recovery following lipophosphoglycan (LPG) stimulation (19). These observations raise the possibility that DCL-patients may harbor less-exhausted T-cell populations capable of responding to PD-1-targeted immunotherapy. Therefore, defining the heterogeneity and differentiation states of exhausted CD4^+^ and CD8^+^ T-cell populations in patients with DCL will be important to determine whether anti-PD-1 immunotherapy represents a viable therapeutic strategy in human leishmaniasis.

In summary, this study demonstrates that variable-dose anti-PD-1 immunotherapy effectively limits experimental chronic leishmaniasis caused by *L. mexicana* at both clinical and parasitological levels by reinvigorating Th1-protective responses. Furthermore, our findings show that PD-1 blockade increases progenitor-like CXCR5^+^TIM-3^-^ and intermediate CXCR5^+^TIM-3^+^ Tex cells during chronic infection, supporting the capacity of ICT to modulate exhausted T-cell responses in the context of a parasitic infection. Importantly, these findings highlight the potential of anti-PD-1 mAb therapy as a promising immunotherapeutic strategy for patients with DCL.

## 4 Limitations and future directions

Although our study demonstrates the therapeutic efficacy of anti-PD-1 immunotherapy during chronic *L. mexicana* infection and provides insights into the Tex populations associated with responsiveness to checkpoint blockade, several limitations should be acknowledged. First, although the variable-dose regimen evaluated in this study showed improved therapeutic efficacy compared with the fixed-dose protocol, the dosing strategy was empirically optimized within this infection model and was not guided by PK/PD analyses. The optimal dose, treatment duration, and timing of PD-1 blockade during chronic parasitic infections remain to be defined. Second, the broader effects of prolonged checkpoint blockade on systemic immune homeostasis and potential immune-related toxicity were not evaluated in this study. Although no overt signs of toxicity or weight loss were observed during treatment, additional studies will be necessary to establish the safety profile of our variable-dosing regimen. Third, the long-term therapeutic efficacy of anti-PD-1 immunotherapy was not assessed, and therefore it remains unclear how long disease progression and parasite control can be sustained after treatment discontinuation. Future longitudinal studies will be necessary to determine the durability of the therapeutic response and whether additional maintenance strategies might be required to preserve infection control over time. Fourth, although our data show that PD-1 blockade is associated with expansion of progenitor-like and intermediate Tex populations, the precise contribution of these subsets to parasite control remains unresolved. Future studies incorporating adoptive transfer approaches, lineage tracing, or subset-specific depletion strategies will be required to determine whether these subsets mediate the therapeutic effects observed following anti-PD-1 therapy. Finally, the heterogeneity of Tex populations in human leishmaniasis remains poorly characterized. Future efforts should therefore prioritize defining the phenotypic and functional landscape of Tex subsets in patients with DCL. Such studies will be important to assess the translational potential and clinical feasibility of checkpoint-targeted immunotherapy for patients with DCL.

## 5 Materials and methods

### 5.1 Animals and ethics statement

Eight- to ten-week-old female C57BL/6 mice were purchased from the pathogen-free animal breeding facility at the Experimental Medicine Research Unit of the Faculty of Medicine, National Autonomous University of Mexico (UNAM). Animals were selected randomly and kept under controlled temperature and light conditions in standard rectangular cages, with food and water ad libitum. All animal procedures were carried out according to the guidelines established by the Official Mexican Standard NOM-062-ZOO-1999 about technical specifications for the production, care and use of laboratory animals, with approval by the Internal Committee for the Care and Use of Laboratory Animals (CICUAL) from the Faculty of Medicine, UNAM registered under the number 024-CIC-2023.

### 5.2 Parasites and infection

*Leishmania mexicana* (MHOM/MX/2011/Lacandona) promastigotes obtained from lesion-derived amastigotes were cultured at 26 °C in M-199 medium, pH 7.2 (with Hank´s salt and L-glutamine without sodium bicarbonate; Sigma-Aldrich) supplemented with 10% heat-inactivated fetal bovine serum (FBS; Gibco, Thermo Fisher Scientific), 2 mM L-glutamine (Gibco, Thermo Fisher Scientific), 4.1 mM sodium bicarbonate (J. T. Baker), 25 mM HEPES (Sigma-Aldrich), 100 U/mL penicillin G and 100 μg/mL streptomycin (both from Sigma-Aldrich) (55). Stationary-phase promastigotes on the 5th day of culture were harvested by centrifugation and washed twice with phosphate buffer saline (PBS), pH 7.4. Mice were subcutaneously infected into the right hind footpad with 1x10^5^ parasites. Lesion development was monitored every 3–4 days by measuring the increase of footpad thickness using a digital micrometer caliper. Freeze-thawed pLAg was obtained from stationary-phase promastigotes culture, as previously described (28).

### 5.3 Anti-PD-1 checkpoint blockade

Anti-mouse PD-1 (CD279) mAb, clone RMP1-14 (Ultra-LEAF Purified, BioLegend) was diluted in 1X PBS and injected intraperitoneally (i.p.) following two different protocols in separate experimental groups. In protocol (1), anti-PD-1 was administered at 100 μg/dose (4.5 mg/kg) twice weekly for 36 days, with a total of 10 doses (1000 μg/mouse), starting at day 52 post-infection. In protocol (2), anti-PD-1 was administered at 250 μg/dose (11.4 mg/kg) every three days for three consecutive doses beginning at day 45 post-infection, followed by a five-day resting period and subsequently five administrations of 100 μg/dose (4.5 mg/kg) every three days (1,250 μg/mouse). Non-treated control animals received PBS, following the corresponding treatment schedules. All mice were weighed weekly and euthanized three days after the final dose of anti-PD-1 mAb.

### 5.4 Parasite load

Parasite burden in footpad lesions was evaluated in digital images captured from histological sections stained with Hematoxylin and Eosin (H&E). Microphotographs were acquired with an AxioCam MRc5 camera using a Carl Zeiss Axio Imager M1 microscope (Carl Zeiss). The FIJI ImageJ Software 1.47v (ImageJ NIH) was used to count the amastigotes present in 16 images captured at 40x magnification, corresponding to 2 mm^2^.

Parasite load in popliteal dLNs was determined by direct counting of amastigotes in a Neubauer hemocytometer under a light microscope at 40x magnification. In brief, footpads and dLNs were excised and homogenized through 40 μm-pore-size cell strainers with PBS using syringe plungers. The resultant homogenate containing free amastigotes was pelleted by centrifugation and adjusted to a known volume prior to parasite count. Remaining intracellular amastigotes were released by passing through 27G x 13 mm needles, repeating the process at least three times before counting.

### 5.5 Cytokines in culture supernatants

Popliteal dLNs were mechanically dissociated through a 40-μm nylon filter and washed twice with PBS. The resultant homogenate was pelleted by centrifugation and resuspended in complete RPMI-1640 medium, pH 7.2 (with L-glutamine, without sodium bicarbonate; Gibco-Invitrogen) containing 2 g/L sodium bicarbonate, 2 mM L-glutamine, 10 mM HEPES, 55 μM 2-mercaptoethanol (Thermo Fisher Scientific), 10% FBS, 100 U/mL penicillin G, and 100 μg/mL streptomycin. Isolated cells were then seeded at a density of 1x10^6^ cells per well in 96-flat bottom plates and stimulated with 5 μg/mL ConA (Sigma-Aldrich) or 10 mg/mL pLAg for 96 h at 37 °C in a 5% CO2 atmosphere. Culture supernatants were collected, clarified by centrifugation and stored at -70 °C until use. Cytokine levels were determined by LEGENDplex bead-based immunoassay using Mouse Th1/Th2 Panel (BioLegend) for flow cytometry, as previously described (28).

### 5.6 Flow cytometry

Freshly isolated cells from dLNs were seeded in 96-well flat-bottom plates at a density of 1x10^6^ cells/well in complete RPMI-1640 medium. For intracellular cytokine detection and degranulation assay, cells were stimulated for 6 h at 37 °C with an activation cocktail containing 50 ng/mL phorbol 12-myristate 13-acetate (PMA) and 1 μg/mL ionomycin calcium salt (both from Sigma-Aldrich), with 2 μM monensin (BioLegend) added during the final 4 h of incubation. For proliferation assay, cells were stimulated with 5 μg/mL ConA for 96 h at 37 °C. The following fluorochrome-conjugated mAbs were used for surface and intracellular staining (all from BioLegend unless otherwise specified): anti-CD3 (145-2C11), anti-CD4 (GK1.5), anti-CD8 (53-6.7), anti-PD-1 (29F.1A12), anti-CXCR5 (QA20A65), anti-TIM-3 (RMT3-23; Tonbo Biosciences), anti-IFN-γ (XMG1.2; Tonbo Biosciences), anti-TNF-α (MP6-XT22), anti-CD107a (1D4B), anti-Granzyme B (GB11), and anti-Ki-67 (16A8). Fixable viability Ghost Dye Violet 510 (Tonbo Biosciences) was used to exclude dead cells. Surface staining was performed for 20 min at 4 °C using an antibody cocktail, followed by fixation with 2% paraformaldehyde for 15 min at 4 °C. After fixation, cells were permeabilized twice with 1X Perm/Wash buffer (BioLegend) and subsequently stained for 30 min at 4 °C with intracellular antibodies diluted in permeabilization solution. Samples were acquired using a Cytek Aurora Spectral cytometer (Cytek Biosciences) and analyzed by FlowJo v.10 Software (Treestar). Gating strategy for Ki-67, IFN-γ, and Granzyme B expressions are shown in **Supplementary Figure 2**.

### 5.7 Immunofluorescence

Footpad tissues were fixed in 4% formaldehyde for 24–72 h, embedded in paraffin, and sections into 3-μm slices to mounted on positively charged glass slides. Tissue sections were deparaffinized by incubating at 60-65 °C for 45 min, followed by a 5 min incubation in xylene and rehydration through a graded series of xylene/ethanol solutions (Xylene50%/Et50%, Et100%, Et80%, Et80%, Et50%, H20). Heat-induced antigen retrieval was performed using 10 mM sodium citrate buffer, pH 6.0 (Sigma-Aldrich), at 90 °C for 20 min. Sections were permeabilized in a solution of 10 mg/mL bovine serum albumin (Sigma-Aldrich), 5% horse serum, 0.02% sodium azide, and 0.3% Triton X-100 (Sigma-Aldrich) for 2 h. Following permeabilization, tissues were incubated overnight with the following fluorochrome-conjugated antibodies (all from BioLegend unless otherwise specified): anti-CD4 (GK1.5), anti-CD8 (53-6.7), anti-PD-1 (29F.1A12), anti-CXCR5 (L138D7), anti-TIM-3 (RMT3-23; Tonbo Biosciences), anti-IFN-γ (XMG1.2), and anti-TNF-α (MP6-XT22) at a 1:50 dilution. Nuclei were counterstained with Hoechst 33258 (1:2000) for 10 min, and slides were mounted with VectaShield Mounting Medium (Vector Laboratories). Three H&E-defined areas of each tissue with pronounced immune cell infiltration were analyzed to determine the percentage of the phenotypes of interest. Micrographs were captured using a Nikon Ti Eclipse inverted confocal microscope (Nikon Corporation) equipped with an A1 system, controlled by NIS Elements v.4.50 software. A 20x (dry, NA 0.75) objective lens was used, with additional magnification (3.4x) through Nyquist´s sampling during image acquisition. Confocal images were analyzed using FIJI ImageJ Software 1.47v.

### 5.8 Statistical analysis

All statistical analyses were performed using GraphPad Prism 8.0.1 (GraphPad Software Inc.). For pairwise comparisons between untreated and treated animals, nonparametric Mann-Whitney U-test was applied in all cases. *P* values < 0.05 were considered statistically significant. Unless otherwise indicated, data are shown as individual values, with each point representing one independently analyzed mouse, together with the median and full range for each experimental group. To provide a quantitative measure of the magnitude and direction of differences observed between experimental groups, effect size was estimated using the Hodges-Lehmann estimator, representing the median of all pairwise differences between groups. Exact confidence intervals (CI) of each difference were reported and calculated, requesting 95% CI for all analyses. CI levels were automatically adjusted by GraphPad Prism to the closest achievable value to the requested 95% for each analysis.

## Data Availability

The original contributions presented in the study are included in the article/**Supplementary Material**. Further inquiries can be directed to the corresponding authors.
