# Supplementary material for: Immunotherapeutic potential of PD-1 blockade in chronic Leishmania mexicana infection through the enhancement of progenitor-like CXCR5+ and intermediate CXCR5+TIM-3+ exhausted T cells
Source: Front Immunol. 2026 Jun 29;17:1827753. doi: 10.3389/fimmu.2026.1827753 (PMC13357283; doi:10.3389/fimmu.2026.1827753)
Supplement: Supplementary file 1 [file DataSheet1.pdf]

Supplementary Figures

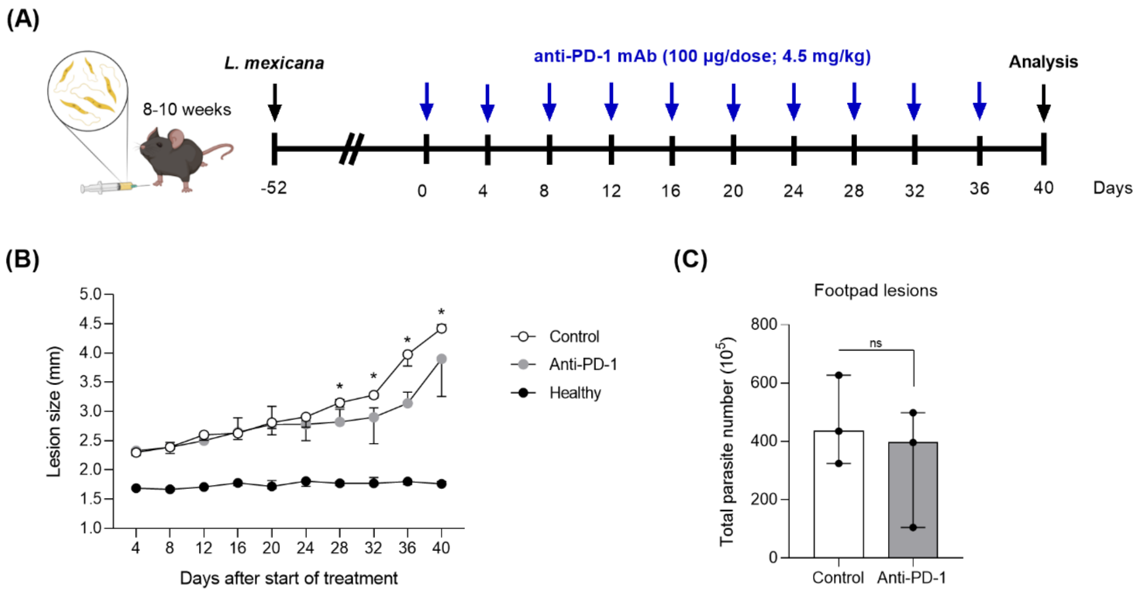

Supplementary Figure 1. Repeated fixed-dose anti-PD-1 therapy modestly reduces lesion size during chronic *L. mexicana* infection. (A) C57BL/6 mice were subcutaneously infected with $1x10^5$ stationary-phase promastigotes and treated intraperitoneally with anti-PD-1 mAb (100 µg/dose), administered twice weekly starting at day 52 post-infection. (B) Lesion progression was monitored by measuring footpad thickness with digital vernier caliper. Each point representing the median value obtained from 3-4 independently analyzed mice, with error bars indicating the full range (min–max). Asterisks indicate significant differences between untreated and anti-PD-1-treated mice. (C) Parasite burden at lesion sites was quantified at the end of treatment. Each dot represents one independently analyzed mouse (n=3-4), and bars indicate the median and full range. All statistical analyses were performed using Mann-Whitney U-test. $*P \leq 0.05$.

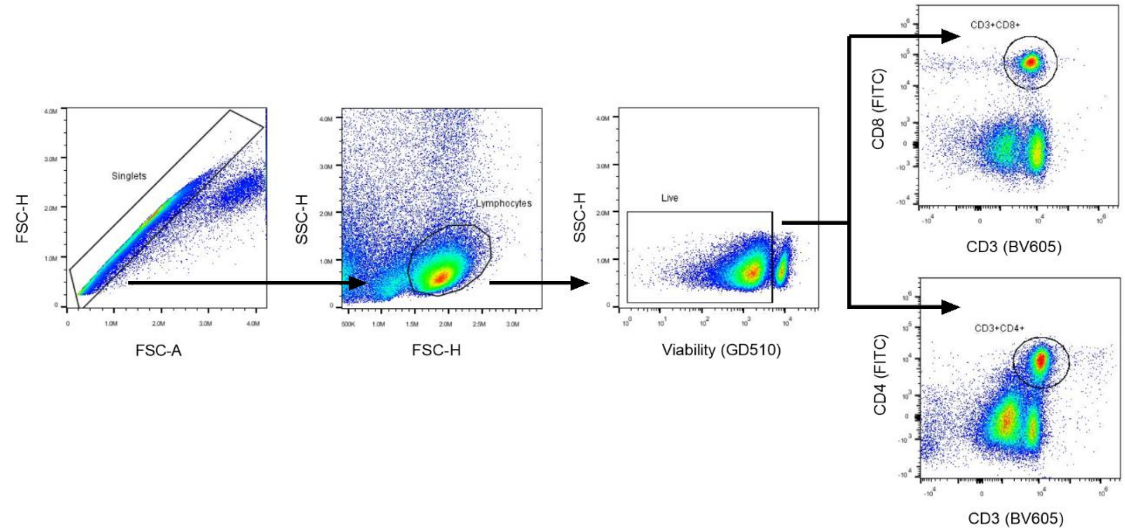

Supplementary Figure 2. Gaiting strategy used flow cytometry analysis shown in Figure 3. Doublets were excluded using forward scatter-height (FSC-H) versus forward scatter-area (FSC-A) plots, and lymphocytes were identified based on side-scatter-height (SSC-H versus FSC-H parameters. Dead cells were excluded by gating on viability dye-negative events. Live cells were subsequently gated on $CD3^{+}CD8^{+}$ or $CD3^{+}CD4^{+}$ cells to evaluate Ki-67, IFN-γ and GrzmB expression.
